# Single-parenthood and health conditions among children receiving public assistance in Japan: a cohort study

**DOI:** 10.1186/s12887-021-02682-4

**Published:** 2021-05-03

**Authors:** Daisuke Nishioka, Junko Saito, Keiko Ueno, Naoki Kondo

**Affiliations:** 1 Department of Medical Statistics Research & Development Center, Osaka Medical and Pharmaceutical University, Daigakumachi 2-7, Takatsuki-shi, Osaka, Japan; 2 Department of Health and Social Behavior, Graduate School of Medicine, The University of Tokyo, Hongo 7-3-1, Bunkyo-ku, Tokyo, 113-0033 Japan; 3 Department of Social Epidemiology, Graduate School of Medicine and School of Public Health, Kyoto University, Kyoto, Japan; 4 Behavioral Science Division, Behavioral Sciences and Survivorship Research Group, Center for Public Health Sciences, National Cancer Center, 5-1-1 Tsukiji, Chuo-ku, Tokyo, 104-0045 Japan

**Keywords:** Poverty, Public assistance, Single-parenthood, Chronic health conditions, Japan

## Abstract

**Background:**

Children’s healthy development is important. While governmental public assistance benefits financially troubled families, it cannot compensate for a lack of social support. Single-parenthood is a health risk factor for children owing to low-income-associated food insecurity and stress. No study has investigated the association between single-parenthood and health status in children from families receiving public assistance. This study aimed to examine the association between single-parent households and children’s health among public assistance recipients in Japan by using linkage data of two municipal public assistance databases and administrative medical assistance data.

**Methods:**

We performed a retrospective cohort study. Public assistance for households below the poverty line ensures income security and medical care. The study population included all children aged 15 or younger availing public assistance in January 2016. We extracted recipients’ sociodemographic factors from January 2016 and identified the incidence of childhood diseases’ diagnosis until December 2016 as the outcome, including 1) acute upper respiratory infections; 2) influenza and pneumonia; 3) injuries, including fractures; 4) intestinal infectious diseases; 5) conjunctivitis; 6) asthma; 7) allergic rhinitis; 8) dermatitis and eczema, including atopic dermatitis; and 9) diseases of the oral cavities, salivary glands, and jaws, such as tooth decay or dental caries.

**Results:**

Among the 573 children, 383 (66.8%) lived in single-parent households. A multivariable Poisson regression, with a robust standard error estimator, showed that single-parenthood is associated with a higher prevalence of asthma (incidence ratio [IR] = 1.62; 95% confidence interval [CI], 1.16–2.26), allergic rhinitis (IR = 1.41; 95% CI, 1.07–1.86), dermatitis and eczema (IR = 1.81; 95% CI, 1.21–2.70), and dental diseases (IR = 1.79; 95% CI, 1.33–2.42) compared to non-single parent households, whereas little association was found between single-parenthood and children’s acute health conditions.

**Conclusions:**

Among public assistance recipients, living in single-parent households may be a risk factor for children’s chronic diseases. The Japanese public assistance system should provide additional social care for single-parent households. Further investigations are necessary using more detailed longitudinal data, including environmental factors, the severity of children’s health conditions, contents of medical treatments, and broader socioeconomic factors.

**Supplementary Information:**

The online version contains supplementary material available at 10.1186/s12887-021-02682-4.

## Introduction

Ensuring healthy physical development of children is an important societal concern [1]. Early social and environmental experiences, as well as certain genetic predispositions, affect children’s health statuses and also influence the development of adaptive behaviors, learning capacities, prospective physical and mental health statuses, and future human resources or economic productivity [2–5]. Despite a strong commitment to the health and development of all children, there are still concerns about poor childhood health outcomes determined by issues within the social determinants of health. In recent studies, single-parenthood has been recognized as a risk factor for the development of chronic health conditions among children [6–13]. Children in single-parent households are seen as a socially vulnerable population, as their families tend to have a lower overall income [6], resulting in food insecurity [14] and other physical conditions that impose a heightened risk for maladaptive health outcomes [6, 10–13]. Furthermore, these children tend to experience a stronger psychological burden [6, 12, 15], as they lack instrumental and emotional social support [6, 15–17].

Although the association between single-parenthood and children’s chronic health conditions has been attributed to financial difficulties or psychosocial stress, previous studies have not identified this mechanism in sufficient detail. Thus, an effective approach aimed at maintaining children’s positive health statuses in single-parent households should still be developed [18, 19]. Furthermore, few studies have focused on the health statuses of children receiving governmental welfare benefits, who are susceptible to several social determinants of health. In Japan, the poverty rate among single-parent households was 50.8% in 2013, which was the highest among all Organization for Economic Cooperation and Development member countries [20, 21]; many children in these households are facing risks associated with public assistance. Using Japanese public assistance data, we may be able to support children’s overall health in these households. To fill the gap in the literature on this topic, this study aimed to examine the association between single-parenthood and children’s chronic health conditions among families receiving governmental public assistance, who were ensured a monthly minimum income protection and were fully exempted from the co-payments for their medical care utilization.

Hence, the objective of this study was to examine the association between children’s health statuses and their household compositions among those living on public assistance by using aggregated public assistance and medical assistance claim data as administered by Japanese municipalities.

## Material and methods

### Data sources

This cohort study included all children aged 15 or younger who were on public assistance in January 2016 from two suburban municipalities in Japan. We observed the participants for a year until December 2016. Public assistance is a governmental welfare program which is eligible to households that live below the poverty line without any assets. The municipal welfare office conducts rigorous means tests (official investigations into households’ financial circumstances) and decides whether or not the households receive the benefits. The Japanese public assistance system is composed of eight assistance categories (i.e., Livelihood, Education, Housing, Medical, Long-term care, Maternity, Occupational, Funeral Assistance), and all recipients can receive all the benefits they need. Households on public assistance are financed by governmental subsidies whereby they receive a monthly minimum income protection. All recipients are allowed to use medical assistance if they have medical-care vouchers (*iryo-ken*) from the welfare office; otherwise, the recipient’s medical payment is supported by other welfare services such as the designated intractable disease assistance or disability assistance.

For baseline data, we used public assistance databases as administered by the welfare offices of the chosen municipalities in January 2016. These databases include information on peoples’ age, sex, number of family members, household composition, nationality, working status, and income including working income, pensions, and disability pensions. These data were collected by staff members working at the welfare office in each municipality. Since this information is required to determine the total applications for public assistance and the amount of each family’s monthly minimum income protection, there were no missing data. Moreover, to obtain the outcome data, we used medical assistance administrative data from January 2016 to December 2016. These data were administered by a management software for the public assistance database to record the amount of municipal medical assistance payment for each recipient, which included the months of the recipients’ medical consultations, the total cost of their medical claims, the total numbers of visits in each month, and their diagnoses. However, this administrative data did not include information on the severity of diseases, contents of medical treatment, flags of the primary diagnosis, suspected diagnosis, cured diagnosis, or date of the diagnosis. This database included medical, diagnosis procedure combination, dental, pharmacy, and visiting nurses claims covered by Japan’s National Health Insurance program [22].

Each municipality aggregated the sociodemographic and medical assistance claim data with the public assistance database using individual identification codes. The welfare offices of the two participating municipalities agreed to provide anonymized public secondary data to the authors via a system company, which provided the management software for the public assistance database used by the municipality welfare offices. Informed consent was waived by the Research Ethics Committee of the Graduate School of Medicine of the University of Tokyo because of encrypted identification numbers. Further, the parents or legal guardians of those in the study population were provided with opportunities to opt out of inclusion in each municipality. The study protocol was approved by the Research Ethics Committee of the Graduate School of Medicine of the University of Tokyo (Approval No: 11503). This study was carried out in accordance with STROBE (Strengthening the Reporting of Observational Studies in Epidemiology) guidelines.

### Measurement and variables

#### Childhood diseases

From the medical assistance claim data, we identified the cumulative incidence of each diagnosis, defined as those who visited medical care institutions for the following diseases once or more during the observation period: 1) acute upper respiratory infections; 2) influenza and pneumonia; 3) injuries, including fractures; 4) intestinal infectious diseases; 5) conjunctivitis; 6) asthma; 7) allergic rhinitis; 8) dermatitis and eczema, including atopic dermatitis; and 9) diseases of the oral cavities, salivary glands, and jaws, such as tooth decay or dental caries. We identified these diagnoses as common medical conditions among children in Japan [23–25]. We regarded the diseases from 1) to 5) as acute health conditions and those from 6) to 9) as chronic health conditions. Each diagnosed health condition listed above was identified using the corresponding International Classification of Disease, Tenth Edition (ICD-10) codes, which are: 1) J00–06, 2) J10–18, 3) S00–99 and T00–14, 4) A00–09, 5) H10, 6) J45–46, 7) J30, 8) L20–30, and 9) K00–06 [26].

#### Household composition

We extracted information on the participants’ household compositions from the database in January 2016. In the Japanese public assistance system, if children live with only a single parent (father or mother only), and not with their grandparents or other caregivers, their household type is defined as a single-parent household. If the children live with any relatives or caregivers other than their single parents, their household status was labeled as other statuses [27]. According to the collected information, we defined a binary variable of living in a single-parent household or not.

#### Covariates

Based on the available evidence, for demographic factors, we used age (continuous), sex (girl/boy), presence of siblings (being an only child or having siblings), and nationality (Japanese or not), as of January 2016. For participants’ socioeconomic characteristics, we used the working statuses of their parents (working or not). We used this variable as a covariate because the working statuses of parents may influence the amount of time that they have available to care for their children. We coded the municipality in which the participants lived as a dummy variable in order to adjust for the unmeasured cultural and environmental characteristics of the two municipalities (A/B). For example, there is an environmental difference in the form of air pollution between Municipality A in Tokyo and Municipality B in Osaka; an air quality index [28] registers Municipality A as “Good” but Municipality B as “Moderate” [29]. In addition, Municipality A is a supportive municipality in Japan for single-parent households and offers more extensive governmental welfare support [30].

### Statistical analysis

First, we described children’s characteristics and the children diagnosed with acute and chronic diseases during the study period. Second, because the proportional hazards of consultations were not constant over the observed period, we identified the one-year-cumulative incidence of the disease diagnoses and compared that information with the child’s household status (single-parent or not). To calculate the cumulative incidence ratio, we performed a univariate Poisson regression analysis and calculated the crude incidence ratio of each diagnosis (IR) and its 95% confidence interval (CI) for each explanatory variable. Third, we performed a multiple Poisson regression analysis to calculate the multivariable-adjusted IR of each disease. The robust standard error estimator was adopted in order to calculate the 95% CI. For a robustness check, we performed a sensitivity analysis, excluding the data of children who stopped receiving public assistance during the study period. Further, previous studies in Japan showed that asthma, allergic rhinitis, and dermatitis and eczema have specific age patterns with regard to their morbidities [31–33]; we thus performed age-stratified analyses on these three diseases, stratifying the age groups into infant/pre-school age (0–5 years old) and school age (6 years old or more). Analyses were performed using the STATA SE Ver.16.2 program for all of the statistical analyses (Stata Corp., College Station, TX, USA).

## Results

We obtained the data of 573 children from families on public assistance. Among them, 290 (50.6%) were boys, 383 (66.8%) lived in single-parent households, and 478 (83.4%) had undergone a medical consultation. During the study period, for acute diseases, 286 (49.9%) were diagnosed with an upper respiratory infection, 219 (38.2%) were diagnosed with a lower respiratory infection, and 50 (8.7%) were diagnosed with an injury. For chronic diseases, 146 (25.4%) were diagnosed with asthma, 185 (32.2%) were diagnosed with allergic rhinitis, 108 (18.8%) were diagnosed with dermatitis and eczema, and 181 (31.6%) were diagnosed with one of the dental diseases (Table 1). Among 383 children living in single-parent households, 111 (29.0%) were diagnosed with asthma, 137 (35.8%) were diagnosed with allergic rhinitis, 83 (21.7%) were diagnosed with dermatitis and eczema, and 141 (36.8%) were diagnosed with dental diseases (Table 1).

**Table 1:** Characteristics of overall, children who had consultation, and children diagnosed with each disease

Character	Category	Total participants (*N* = 573) N (%)	Having medical consultation (*n* = 478) n, % for N	Asthma (*n* = 146) n, % for N	Allergic rhinitis (*n* = 185) n, % for N	Dermatitis and eczema (*n* = 108) n, % for N	Dental diseases (*n* = 181) n, % for N
Age	Mean (SD)	9.7 (4.3)	9.4 (4.2)	8.3 (4.2)	9.5 (4.0)	7.8 (4.7)	9.5 (3.7)
Sex							
	Girl	283 (49.4)	225, 79.5%	67, 23.7%	82, 29.0%	47, 16.6%	93, 32.9%
	Boy	290 (50.6)	253, 87.2%	79, 27.2%	103, 35.5%	61, 21.0%	88, 30.3%
Presence of siblings							
	Only child	207 (36.1)	179, 86.5%	56, 27.1%	72, 34.8%	43, 20.8%	72, 34.8%
	Have siblings	366 (63.9)	299, 81.7%	90, 24.6%	113, 30.9%	65, 17.8%	109, 29.8%
Single-parenthood							
	No	190 (33.2)	154, 81.1%	35, 18.4%	48, 25.3%	25, 13.2%	40, 21.1%
	Yes	383 (66.8)	324, 84.6%	111, 29.0%	137, 35.8%	83, 21.7%	141, 36.8%
Parent’s work							
	No	268 (46.8)	225, 84.0%	81, 30.2%	95, 35.4%	57, 21.3%	90, 33.6%
	Yes	305 (53.2)	253, 83.0%	65, 21.3%	90, 29.5%	51, 16.7%	91, 29.8%
Nationality							
	Japanese	534 (93.2)	449, 84.1%	140, 26.2%	175, 32.8%	102, 19.1%	162, 30.3%
	Others	39 (6.8)	29, 74.4%	6, 15.4%	10, 25.6%	6, 15.4%	19, 48.7%
Municipality							
	A	404 (70.5)	327, 80.9%	103, 25.5%	112, 27.7%	79, 19.6%	121, 30.0%
	B	169 (29.5)	151, 89.3%	43, 25.4%	73, 43.2%	29, 17.2%	60, 35.5%
		URI (*n* = 286) n, % for N	Influenza/ pneumonia (*n* = 219) n, % for N	Intestinal infections (*n* = 101) n, % for N	Conjunctivitis (*n* = 102) n, % for N	Injury (*n* = 50) n, % for N	
Age	Mean (SD)	8.7 (4.4)	8.6 (4.1)	8.5 (3.9)	8.9 (4.1)	10.9 (2.8)	
Sex							
	Girl	149, 52.7%	99, 35.0%	42, 14.8%	49, 17.3%	19, 6.7%	
	Boy	137, 47.2%	120, 41.4%	59, 20.3%	53, 18.3%	31, 10.7%	
Presence of siblings							
	Only child	110, 53.1%	81, 39.1%	39, 18.8%	45, 21.7%	17, 8.2%	
	Have siblings	176, 48.1%	138, 37.7%	62, 16.9%	57, 15.6%	33, 9.0%	
Single parenthood							
	No	83, 43.7%	73, 38.4%	30, 15.8%	29, 15.3%	17, 8.9%	
	Yes	203, 53.0%	146, 38.1%	71, 18.5%	73, 19.1%	33, 8.6%	
Parent’s work							
	No	141, 52.6%	107, 39.9%	44, 16.4%	40, 14.9%	23, 8.6%	
	Yes	145, 47.5%	112, 36.7%	57, 18.7%	62, 20.3%	27, 8.9%	
Nationality							
	Japanese	270, 50.6%	210, 39.3%	96, 18.0%	95, 17.8%	46, 8.6%	
	Others	16, 41.0%	9, 23.1%	5, 12.8%	7, 17.9%	4, 10.3%	
Municipality							
	A	193, 47.8%	143, 35.4%	81, 20.0%	73, 18.1%	20, 5.0%	
	B	93, 55.0%	76, 45.0%	20, 11.8%	29, 17.2%	30, 17.8%	

*SD* Standard deviation; *URI* Upper respiratory infections

The results of the univariate Poisson regression analysis showed that living in a single-parent household was associated with a higher incidence of chronic disease diagnoses compared to not living in a single-parent household (Table S1). The multivariate Poisson regression analysis revealed that children living in single-parent households had an increased incidence of diagnosis in asthma (IR = 1.62; 95% CI, 1.16–2.26), allergic rhinitis (IR = 1.41; 95% CI, 1.07–1.86), dermatitis and eczema (IR = 1.81; 95% CI, 1.21–2.70), and dental diseases (IR = 1.79; 95% CI, 1.33–2.42) compared to those not living in single-parent households (Table 2). However, there was little association between children’s household compositions and acute health conditions. (Table 2). The results of a sensitivity analysis, excluding a total of 31 children who stopped receiving public assistance during the study period, showed similar results (Table S2). Further, the age-stratified analysis showed similar results; children living in single-parent households had a higher incidence of diagnoses of asthma, allergic rhinitis, and dermatitis and eczema when compared to non-single-parent household (Table S3).

**Table 2:** Adjusted incidence ratios (IR) and 95% confidence intervals (CI) for diagnosis of acute and chronic diseases among children receiving public assistance

	Asthma	Allergic rhinitis	Dermatitis/ eczema	Dental diseases	URI	Influenza/ pneumonia	Intestinal infections	Conjunc-tivitis	Injury
	IR (95% CI)	IR (95% CI)	IR (95% CI)	IR (95% CI)	IR (95% CI)	IR (95% CI)	IR (95% CI)	IR (95% CI)	IR (95% CI)
*Explanatory* variable									
Single-parenthood									
No	Ref	Ref	Ref	Ref	Ref	Ref	Ref	Ref	Ref
Yes	1.62 (1.16–2.26)	1.41 (1.07–1.86)	1.81 (1.21–2.70)	1.79 (1.33–2.42)	1.25 (1.03–1.50)	1.02 (0.82–1.27)	1.36 (0.92–2.01)	1.24 (0.84–1.84)	0.98 (0.58–1.67)
*Covariates*									
Age									
by 1 year	0.93 (0.91–0.96)	1.00 (0.97–1.03)	0.89 (0.86–0.93)	0.99 (0.97–1.02)	0.95 (0.94–0.97)	0.95 (0.93–0.97)	0.86 (0.83–0.90)	0.97 (0.93–1.01)	1.10 (1.04–1.17)
Sex									
Girl	Ref	Ref	Ref	Ref	Ref	Ref	Ref	Ref	Ref
Boy	1.15 (0.87–1.52)	1.17 (0.92–1.48)	1.29 (0.93–1.78)	0.90 (0.71–1.15)	1.04 (0.89–1.22)	1.14 (0.93–1.41)	1.44 (1.02–2.04)	0.90 (0.64–1.27)	1.40 (0.81–2.41)
Presence of siblings									
Only child	Ref	Ref	Ref	Ref	Ref	Ref	Ref	Ref	Ref
Have siblings	0.83 (0.62–1.12)	0.89 (0.69–1.15)	0.72 (0.51–1.02)	0.88 (0.68–1.13)	0.82 (0.69–0.97)	0.85 (0.68–1.06)	0.75 (0.52–1.08)	0.75 (0.51–1.09)	1.28 (0.7–2.31)
Parent’s work									
No	Ref	Ref	Ref	Ref	Ref	Ref	Ref	Ref	Ref
Yes	0.83 (0.62–1.11)	0.89 (0.69–1.14)	0.98 (0.69–1.40)	0.91 (0.71–1.17)	1.02 (0.86–1.21)	1.05 (0.84–1.30)	0.74 (0.49–1.13)	0.62 (0.42–0.91)	0.89 (0.5–1.57)
Nationality									
Japanese	Ref	Ref	Ref	Ref	Ref	Ref	Ref	Ref	Ref
Others	0.69 (0.31–1.50)	0.91 (0.52–1.60)	0.91 (0.43–1.91)	1.81 (1.27–2.57)	0.87 (0.6–1.27)	0.63 (0.36–1.11)	0.86 (0.6–1.23)	1.15 (0.57–2.31)	1.66 (0.6–4.64)
Municipality									
A	Ref	Ref	Ref	Ref	Ref	Ref	Ref	Ref	Ref
B	0.94 (0.69–1.27)	1.53 (1.21–1.93)	0.82 (0.57–1.17)	1.26 (0.98–1.63)	1.14 (0.96–1.35)	1.22 (0.99–1.51)	0.52 (0.34–0.79)	0.95 (0.65–1.40)	3.55 (2.05–6.16)

*URI* Upper respiratory infections

## Discussion

Among the participating children living in families on governmental public assistance in Japan, the incidence of chronic diseases such as asthma, allergic rhinitis, dermatitis and eczema, and dental diseases was higher among those living in single-parent households compared to non-single-parent households. However, there was little association between children’s acute health conditions and living within a single-parent household. This is the first study to identify children’s health statuses among those on public assistance in Japan. Further, an important strength of this study is that, using existing national standardized databases of people receiving public assistance without any missing data, we could examine the association between children’s morbidities and the household compositions of socially vulnerable people, which are usually considered difficult to reach using a standard social survey.

Our findings were consistent with those of recent studies [10, 13, 34, 35]. Moncrief et al. reported a possible association of living in single-parent households and the development of childhood asthma in the United States [13], with this phenomenon being driven by underlying low household incomes among single-parent homes. Our study, thus, provides new evidence supporting the notion that single-parenthood remains a risk for children’s health statuses even among families whose minimum income protection and healthcare access are financially ensured by the government.

### Interpretation

There are possible explanations for the higher incidence of these chronic diseases in children living in single-parent households compared to those in other household types. First, because single parents do not have partners that share financial resources and material goods within the households and rear children together, they are more likely to be in material poverty and have psychological stresses that influence all household members, including their children [17, 36–38]. For example, smoking, a typical stress-relieving behavior, is higher among single-parent households than non-single-parent households in Japan [39]. In Japan, the poverty rate of single parent households is extremely high (over 50%) [21]; even among those receiving public assistance, the incomes of single-parent households are lower than those of their counterparts. Children’s chronic health conditions may develop due to poverty before they receive public assistance. After they have received public assistance benefits, they may have been able to see doctors for their chronic health conditions.

Furthermore, Mullins et al. identified an association between being a single parent and people’s parenting capacities, suggesting that children living in single-parent households may not receive enough care, resulting in their increased morbidity of chronic health conditions [36]. Several studies have reported that parental psychosocial stress increases the risk of their children developing asthma and allergic diseases [40–44].

On the other hand, there is a possibility that children’s health status in single parent household does not differ from that of other households. Our results might be derived from single parents’ medical consultation behavior; single parents may notice their children’s diseases more often than those in non-single parent households. Children’s disease symptoms in single-parent households may receive greater attentions owing to the increased level of affection or overprotection received from single parents. Children in single-parent households also use more social resources, such as after school care, given their parent’s work conditions [45], which may also result in an increased detection of any disease symptoms.

Our results showing little association between acute symptoms and household types support the hypothesis that children in single parent household have a higher prevalence of chronic conditions. We can consider medical consulting behavior is likely consistent between the different household types if children develop more noticeable acute symptoms in either type of household.

### Implications

Our results suggest important policy implications. Ameliorating the health conditions of children living in single-parent households on public assistance can be achieved by closer monitoring in the community. For example, regarding public assistance policies, the government has launched a health management program for people on public assistance that is  mandated for municipality governments in 2021 [27]. This program’s purpose includes supporting the healthy lives of children on public assistance. Given the findings of this study, those activities may provide additional care for children living in single-parent households. Further, according to the policy based on the Law to Promote Measures against Child Poverty, the Japanese government recommends supporting children in need by strengthening community governance within welfare offices, healthcare institutes, and nurseries or schools [46]. Our study indicates that the policy should provide more focused support for single-parent households on public assistance to ensure the healthy development of children. On the other hand, cooperation between formal services and informal support for single-parent households is also important. For example, there is a nationwide network of voluntary and/or non-profit organizations that support single-parent households in the community (e.g., single mothers forum) [47].

### Limitations

There are several limitations to this study. First, although we analyzed cohort data, there is still the possibility of reverse causation. For instance, if children have severe chronic diseases, especially in single-parent households, their parents might not be able to work as they have to care for their children, resulting in financial difficulties and the subsequent receipt of public assistance. Second, the variables used in our analysis were limited; important unmeasured factors that lead to chronic diseases including other social background variables such as social relationships, environmental factors (e.g., the smoking habits of family members), and medical history (e.g., the severity of diseases, or contents of medical treatment) were not considered in this study. Because of the availability of the medical assistance data, we could not judge whether a recipient’s healthcare visit was for a preventive/evaluation purpose or because of an exacerbation. Third, because these data did not include any flags of primary diagnosis, suspected diagnosis, or cured diagnosis, we could not determine if the children had newly diagnosed diseases. The medical assistance data we used can also code diagnoses even though it is not clinically confirmed. In Japan, to claim insurance payments, doctors must report the name of disease diagnoses including “suspected” diseases. This process might result in an overestimation of the incidence of the diagnoses in this study. Further, because the administrative medical assistance data did not include the claim of payment from other welfare services that were applied for patients with designated intractable diseases or disabilities assistance, we might have misclassified the incidence of diagnosis as no incidence. Fourth, this study’s generalizability could be limited. Because this study only covered two suburban municipalities in Tokyo and Osaka, it may not represent the characteristics of Japan as a whole. For example, unobserved municipality characteristics, including differences in the infrastructure’s services available for single-parent households in each municipality, may influence the findings of this study.

## Conclusions

Children in single-parent households among public assistance recipients in Japan, experience a higher incidence of chronic health conditions such as asthma, allergic rhinitis, dermatitis, and dental diseases, whereas no association was found between single-parenthood and children’s acute health conditions. These results may be explained by the unfavorable living conditions and increased psychosocial stress of this family type, suggesting that the present public assistance system in Japan needs to provide additional social support for single-parent families. Further investigation using more detailed longitudinal information, such as environmental factors, the severity of children’s health conditions, the content of any received treatments, and broader socioeconomic factors is still required.

## Supplementary Information

**Additional file 1. **Single-parenthood and health conditions among children receiving public assistance in Japan: A cohort study. **Table S1.** Crude incidence ratios (IR) and 95% confidence intervals (CI) for diagnosis of acute and chronic diseases among children receiving public assistance. **Table S2.** Adjusted incidence ratios (IR) and 95% confidence intervals (CI) for diagnosis of acute and chronic diseases among children receiving public assistance after excluding children stopped receiving the public assistance. **Table S3.** Adjusted incidence ratios (IR) and 95% confidence intervals (CI) for diagnosis of asthma, allergic rhinitis, and dermatitis among children receiving public assistance, stratified by age.

## Data Availability

The data used in this study were obtained from the participating municipalities in Japan; however, there are restrictions regarding the availability of these data, which were used under license for the current study, and are thus not publicly available. The data are, however, available from the authors upon reasonable request and with the permission of the municipalities.

## Abbreviations

IR: Incidence ratio
CI: Confidence interval
