# Supplementary material for: Single-parenthood and health conditions among children receiving public assistance in Japan: a cohort study
Source: BMC Pediatr. 2021 May 3;21:214. doi: 10.1186/s12887-021-02682-4 (PMC8091550; doi:10.1186/s12887-021-02682-4)
Supplement: Supplementary file 1 — Additional file 1. Single-parenthood and health conditions among children receiving public assistance in Japan: A cohort study. Table S1. Crude incidence ratios (IR) and 95% confidence intervals (CI) for diagnosis of acute and chronic diseases among children receiving public assistance. Table S2. Adjusted incidence ratios (IR) and 95% confidence intervals (CI) for diagnosis of acute and chronic diseases among children receiving public assistance after excluding children stopped receiving the public assistance. Table S3. Adjusted incidence ratios (IR) and 95% confidence intervals (CI) for diagnosis of asthma, allergic rhinitis, and dermatitis among children receiving public assistance, stratified by age. [file 12887_2021_2682_MOESM1_ESM.docx]

**Additional files**

Single-parenthood and health conditions among children receiving public assistance in Japan: A cohort study

Daisuke Nishioka^ab^, Junko Saito^c^, Keiko Ueno^ab^, Naoki Kondo^ab*^

^a^ Department of Health and Social Behavior, Graduate School of Medicine, The University of Tokyo, Hongo 7-3-1, Bunkyo-ku, Tokyo, 113-0033, Japan

^b^ Department of Social Epidemiology and Global Health, Graduate School of Medicine and School of Public Health, Kyoto University, Kyoto, Japan

^c^ Behavioral Science Division, Behavioral Sciences and Survivorship Research Group, Center for Public Health Sciences, National Cancer Center, 5-1-1 Tsukiji, Chuo-ku, Tokyo, 104-0045, Japan

| Table.S1 Crude incidence ratios (IR) and 95% confidence intervals (CI) for diagnosis of acute and chronic diseases among children receiving public assistance. | | | | | | | | | | | | |
| --- | --- | --- | --- | --- | --- | --- | --- | --- | --- | --- | --- | --- |
|  |  |  | Asthma | Allergic rhinitis | Dermatitis and eczema | Dental diseases | URI | Influenza/ pneumonia | Intestinal infections | Conjuncti-  vitis | Injury |  |
|  |  |  | IR  (95% CI) | IR  (95% CI) | IR  (95% CI) | IR  (95% CI) | IR  (95% CI) | IR  (95% CI) | IR  (95% CI) | IR  (95% CI) | IR  (95% CI) |  |
| Age | by 1 year |  | 0.94  (0.91-0.97) | 1.00  (0.97-1.03) | 0.91 (0.87-0.94) | 1.00  (0.97-1.03) | 0.96  (0.94-0.98) | 0.95  (0.93-0.98) | 0.87  (0.84-0.9) | 0.97  (0.93-1.01) | 1.10  (1.04-1.16) |  |
| Sex |  |  |  |  |  |  |  |  |  |  |  |  |
|  | Girl |  | Ref | Ref | Ref | Ref | Ref | Ref | Ref | Ref | Ref |  |
|  | Boy |  | 1.15 (0.87-1.52) | 1.23 (0.96-1.56) | 1.27  (0.90-1.79) | 0.92  (0.73-1.18) | 1.06 (0.90-1.25) | 1.18  (0.96-1.46) | 1.37  (0.96-1.97) | 0.9  (0.63-1.28) | 1.59  (0.92-2.75) |  |
| Presence of siblings | |  |  |  |  |  |  |  |  |  |  |  |
|  | Only child |  | Ref | Ref | Ref | Ref | Ref | Ref | Ref | Ref | Ref |  |
|  | Have siblings |  | 0.91  (0.68-1.21) | 0.89  (0.70-1.13) | 0.85  (0.60-1.21) | 0.86  (0.67-1.09) | 0.90  (0.77-1.07) | 0.96  (0.78-1.19) | 0.90  (0.63-1.29) | 0.72 (0.5-1.02) | 1.09 (0.62-1.92) |  |
| Single parenthood | | |  |  |  |  |  |  |  |  |  |  |
|  | No |  | Ref | Ref | Ref | Ref | Ref | Ref | Ref | Ref | Ref |  |
|  | Yes |  | 1.57 (1.12-2.21) | 1.42 (1.07-1.87) | 1.65 (1.09-2.49) | 1.75 (1.29-2.37) | 1.21 (1.01-1.46) | 0.99 (0.80-1.24) | 1.17 (0.79-1.73) | 1.25 (0.84-1.85) | 0.96 (0.55-1.68) |  |
| Working status of family members | | |  |  |  |  |  |  |  |  |  |  |
| Not working | |  | Ref | Ref | Ref | Ref | Ref | Ref | Ref | Ref | Ref |  |
| Working | |  | 0.71 (0.53-0.94) | 0.83  (0.66-1.06) | 0.79  (0.56-1.11) | 0.89  (0.70-1.13) | 0.90  (0.77-1.06) | 0.92  (0.75-1.13) | 0.68  (0.47-0.97) | 0.57  (0.39-0.81) | 1.03  (0.61-1.76) |  |
| Nationality | |  |  |  |  |  |  |  |  |  |  |  |
| Japanese | |  | Ref | Ref | Ref | Ref | Ref | Ref | Ref | Ref | Ref |  |
| Others | |  | 0.59 (0.28-1.24) | 0.78  (0.45-1.35) | 0.81  (0.38-1.72) | 1.61  (1.14-2.27) | 0.81  (0.55-1.19) | 0.59  (0.33-1.05) | 0.71  (0.31-1.65) | 1.01  (0.50-2.02) | 1.19  (0.45-3.14) |  |
| Municipality | |  |  |  |  |  |  |  |  |  |  |  |
|  | A |  | Ref | Ref | Ref | Ref | Ref | Ref | Ref | Ref | Ref |  |
|  | B |  | 1.00 (0.73-1.36) | 1.56 (1.23-1.97) | 0.88 (0.6-1.29) | 1.19 (0.92-1.53) | 1.15 (0.97-1.37) | 1.27 (1.03-1.57) | 0.59 (0.37-0.93) | 0.95 (0.64-1.4) | 3.58 (2.09-6.13) |  |

SD Standard deviation, URI Upper respiratory infections.

| Table.S2 Adjusted incidence ratios (IR) and 95% confidence intervals (CI) for diagnosis of acute and chronic diseases among children receiving public assistance after excluding children stopped receiving the public assistance. | | | | | | | | | | | | | |
| --- | --- | --- | --- | --- | --- | --- | --- | --- | --- | --- | --- | --- | --- |
|  | |  |  | Asthma | Allergic rhinitis | Dermatitis/eczema | Dental diseases | URI | Influenza/ pneumonia | Intestinal infections | Conjunc-tivitis | Injury |  |
|  | |  |  | IR  (95% CI) | IR  (95% CI) | IR  (95% CI) | IR  (95% CI) | IR  (95% CI) | IR  (95% CI) | IR  (95% CI) | IR  (95% CI) | IR  (95% CI) |  |
| *Explanatory variable* | | | |  |  |  |  |  |  |  |  |  |  |
| Single-parenthood | | |  |  |  |  |  |  |  |  |  |  |  |
|  | | No |  | Ref | Ref | Ref | Ref | Ref | Ref | Ref | Ref | Ref |  |
|  | | Yes |  | 1.65 (1.18-2.31) | 1.39  (1.06-1.82) | 1.85  (1.23-2.78) | 1.79  (1.33-2.43) | 1.22  (1.02-1.47) | 1.02  (0.82-1.26) | 1.32  (0.90-1.94) | 1.26  (0.85-1.86) | 0.95  (0.55-1.65) |  |
| *Covariates* | | |  |  |  |  |  |  |  |  |  |  |  |
| Age | | by 1year |  | 0.94  (0.91-0.97) | 1.00  (0.97-1.03) | 0.89  (0.86-0.93) | 0.99  (0.96-1.02) | 0.96  (0.94-0.97) | 0.95  (0.93-0.97) | 0.86  (0.83-0.89) | 0.97  (0.93-1.01) | 1.10  (1.04-1.17) |  |
| Sex | |  |  |  |  |  |  |  |  |  |  |  |  |
|  | | Girl |  | Ref | Ref | Ref | Ref | Ref | Ref | Ref | Ref | Ref |  |
|  | | Boy |  | 1.14  (0.86-1.50) | 1.15  (0.91-1.45) | 1.26  (0.91-1.75) | 0.87  (0.68-1.11) | 1.05  (0.89-1.23) | 1.14  (0.92-1.40) | 1.41  (1.00-2.00) | 0.90  (0.64-1.27) | 1.41  (0.80-2.48) |  |
| Presence of siblings | | |  |  |  |  |  |  |  |  |  |  |  |
|  | | Only child |  | Ref | Ref | Ref | Ref | Ref | Ref | Ref | Ref | Ref |  |
|  | | Have siblings |  | 0.86  (0.64-1.16) | 0.94  (0.73-1.21) | 0.75  (0.53-1.06) | 0.87  (0.68-1.11) | 0.82  (0.70-0.97) | 0.86  (0.69-1.07) | 0.75  (0.52-1.08) | 0.77  (0.53-1.12) | 1.22  (0.66-2.23) |  |
| Parent’s work | | | |  |  |  |  |  |  |  |  |  |  |
|  | | No |  | Ref | Ref | Ref | Ref | Ref | Ref | Ref | Ref | Ref |  |
|  | | Yes |  | 0.85  (0.64-1.14) | 0.91  (0.71-1.17) | 0.97  (0.68-1.38) | 0.95  (0.74-1.23) | 1.06  (0.90-1.25) | 1.08  (0.87-1.33) | 0.88  (0.61-1.27) | 0.64  (0.44-0.93) | 0.83  (0.46-1.49) |  |
| Nationality | | |  |  |  |  |  |  |  |  |  |  |  |
|  | Japanese | |  | Ref | Ref | Ref | Ref | Ref | Ref | Ref | Ref | Ref |  |
|  | Others | |  | 0.65  (0.30-1.43) | 0.87  (0.49-1.53) | 0.89  (0.42-1.86) | 1.75  (1.23-2.49) | 0.83  (0.57-1.22) | 0.6  (0.34-1.06) | 0.79  (0.34-1.84) | 1.08  (0.53-2.17) | 1.69  (0.60-4.75) |  |
| Municipality | | |  |  |  |  |  |  |  |  |  |  |  |
|  | | A |  | Ref | Ref | Ref | Ref | Ref | Ref | Ref | Ref | Ref |  |
|  | | B |  | 0.92  (0.68-1.25) | 1.50  (1.19-1.9) | 0.80  (0.56-1.16) | 1.28  (0.99-1.65) | 1.14  (0.96-1.34) | 1.20  (0.97-1.48) | 0.51  (0.34-0.78) | 0.91  (0.62-1.33) | 3.46  (1.95-6.13) |  |

URI Upper respiratory infections.

| Table.S3 Adjusted incidence ratios (IR) and 95% confidence intervals (CI) for diagnosis of asthma, allergic rhinitis, and dermatitis among children receiving public assistance, stratified by age. | | | | | | | |
| --- | --- | --- | --- | --- | --- | --- | --- |
|  |  | Preschool children (0 to 5 years old) | | | School children (6 years old or more) | | |
|  |  | Asthma | Allergic rhinitis | Dermatitis | Asthma | Allergic rhinitis | Dermatitis |
|  |  | IR (95% CI) | IR (95% CI) | IR (95% CI) | IR (95% CI) | IR (95% CI) | IR (95% CI) |
| *Explanatory variable* | |  |  |  |  |  |  |
| Single parent household | |  |  |  |  |  |  |
|  | No | Ref | Ref | Ref | Ref | Ref | Ref |
|  | Yes | 1.71 (1.01-2.87) | 1.62 (0.87-3.04) | 1.46 (0.83-2.55) | 1.51 (0.98-2.33) | 1.32 (0.97-1.81) | 1.46 (0.83-2.55) |
| *Covariates* |  |  |  |  |  |  |  |
| Age | by 1 year | 0.99 (0.90-1.10) | 1.01 (0.89-1.14) | 0.95 (0.86-1.05) | 0.92 (0.86-0.99) | 0.97 (0.92-1.02) | 0.95 (0.86-1.05) |
| Sex |  |  |  |  |  |  |  |
|  | Girl | Ref | Ref | Ref | Ref | Ref | Ref |
|  | Boy | 1.50 (0.98-2.31) | 1.27 (0.75-2.16) | 1.12 (0.71-1.76) | 1.01 (0.71-1.45) | 1.14 (0.88-1.49) | 1.12 (0.71-1.76) |
| Presence of siblings | |  |  |  |  |  |  |
|  | Only child | Ref | Ref | Ref | Ref | Ref | Ref |
|  | Have siblings | 0.72 (0.48-1.08) | 0.85 (0.47-1.55) | 0.74 (0.45-1.22) | 0.90 (0.60-1.33) | 0.88 (0.67-1.17) | 0.74 (0.45-1.22) |
| Parent's work | |  |  |  |  |  |  |
|  | No | Ref | Ref | Ref | Ref | Ref | Ref |
|  | Yes | 0.83 (0.52-1.32) | 0.82 (0.46-1.45) | 0.93 (0.57-1.53) | 0.84 (0.58-1.22) | 0.92 (0.70-1.21) | 0.93 (0.57-1.53) |
| Nationality |  |  |  |  |  |  |  |
|  | Japanese | Ref | Ref | Ref | Ref | Ref | Ref |
|  | Others | N/A | N/A | 0.82 (0.32-2.12) | 0.94 (0.44-2.00) | 1.06 (0.61-1.83) | 0.82 (0.32-2.12) |
| Municipality |  |  |  |  |  |  |  |
|  | A | Ref | Ref | Ref | Ref | Ref | Ref |
|  | B | 0.84 (0.54-1.31) | 1.71 (1.02-2.87) | 0.62 (0.34-1.11) | 1.02 (0.68-1.51) | 1.50 (1.14-1.95) | 0.62 (0.34-1.11) |
